# Supplementary material for: Metabolomics Analysis Revealed Significant Metabolic Changes in Brain Cancer Cells Treated with Paclitaxel and/or Etoposide
Source: Int J Mol Sci. 2022 Nov 11;23(22):13940. doi: 10.3390/ijms232213940 (PMC9693830; doi:10.3390/ijms232213940)
Supplement: Supplementary file 1 [file ijms-23-13940-s001.zip › ijms-2016398-supplementary.pdf]

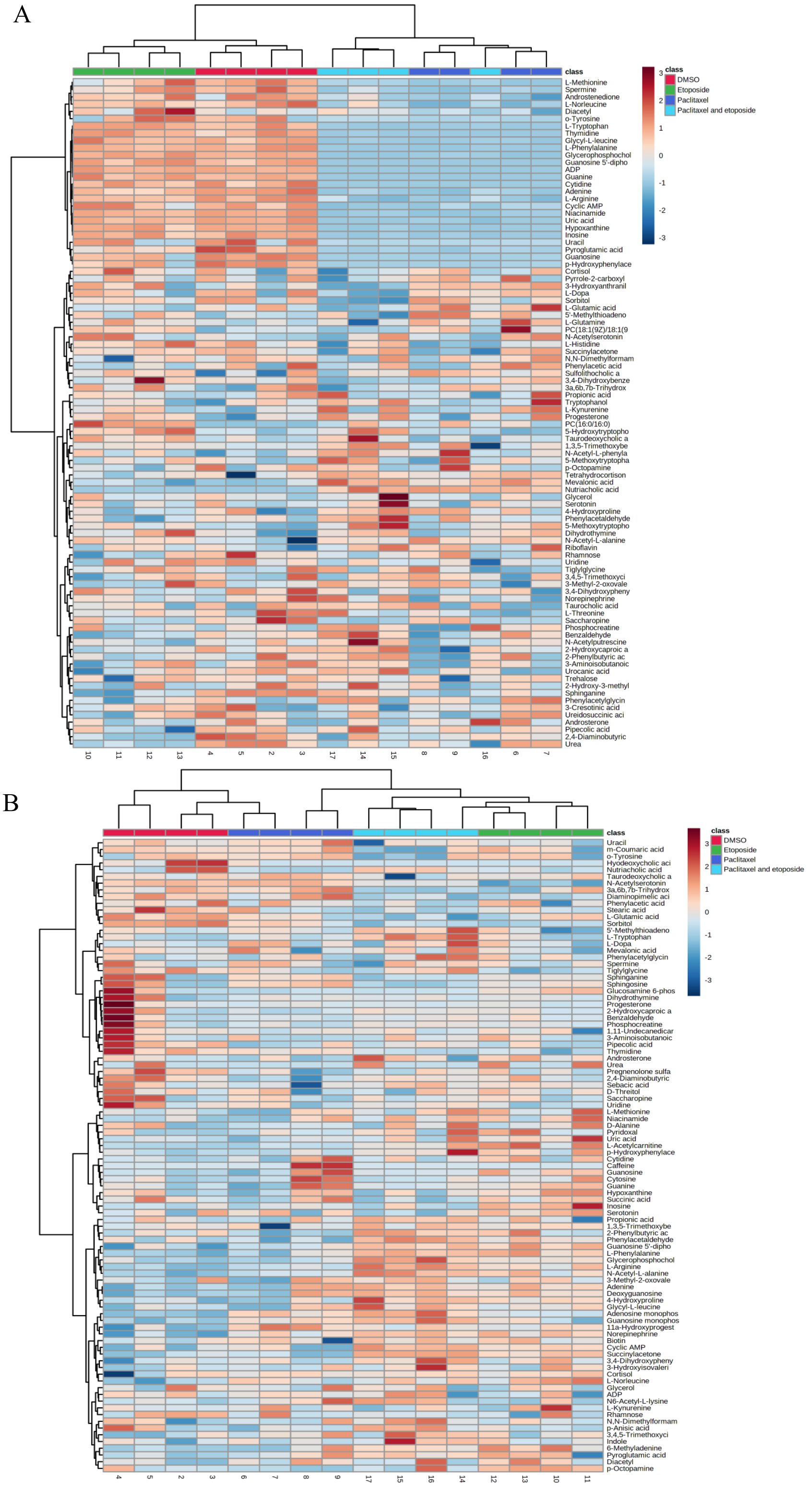

Figure S1. Heat Map for (A) U87 cell line and (B) U373 cell line.

Table S1. ANOVA for U87 and U373 cell lines

ANOVA for 87 cell line treated with paclitaxel 4.2 nM and/or etoposide 10 µg/mL				
	f.value	p.value	-log10(p)	FDR
**ADP**	458.21	1.2274E-12	11.911	1.1292E-10
**Glycerophosphocholine**	320.17	1.0296E-11	10.987	4.7361E-10
**Uric acid**	270.97	2.7619E-11	10.559	6.361E-10
**Niacinamide**	270.9	2.7656E-11	10.558	6.361E-10
**L-Phenylalanine**	178.37	3.2341E-10	9.4902	5.9508E-09
**Guanine**	132.49	1.8346E-09	8.7365	2.7225E-08
**Hypoxanthine**	129.75	2.0715E-09	8.6837	2.7225E-08
**L-Tryptophan**	125.33	2.533E-09	8.5964	2.913E-08
**Adenine**	96.301	1.1594E-08	7.9358	1.1852E-07
**Inosine**	92.872	1.4276E-08	7.8454	1.3134E-07
**Guanosine 5'-diphosphate**	90.065	1.7021E-08	7.769	1.4235E-07
**Glycyl-L-leucine**	66.703	9.3795E-08	7.0278	6.7234E-07
**Thymidine**	66.552	9.5004E-08	7.0223	6.7234E-07
**L-Arginine**	57.115	2.2397E-07	6.6498	1.4718E-06
**Nutriacholic acid**	54.112	3.0257E-07	6.5192	1.8558E-06
**Cytidine**	52.001	3.7733E-07	6.4233	2.1696E-06
**Pyroglutamic acid**	30.784	6.4383E-06	5.1912	3.4843E-05
**Guanosine**	23.978	2.3461E-05	4.6297	0.00011991
**Cyclic AMP**	20.864	4.7199E-05	4.3261	0.00022854
**L-Norleucine**	20.62	5.0034E-05	4.3007	0.00023016
**Spermine**	19.218	7.0785E-05	4.1501	0.00031011
**p-Hydroxyphenylacetic acid**	18.688	8.1138E-05	4.0908	0.0003393
**L-Methionine**	17.739	0.00010444	3.9811	0.00041775
**Mevalonic acid**	14.938	0.00023559	3.6278	0.0009031
**PC(16:0/16:0)**	12.743	0.00048625	3.3131	0.0017894
**Sphinganine**	12.355	0.0005578	3.2535	0.0019737
**Androstenedione**	12.027	0.00062824	3.2019	0.0021407
**L-Glutamic acid**	9.7438	0.0015436	2.8115	0.0050718
**Urea**	8.0001	0.0033984	2.4687	0.010781
**5-Hydroxytryptophol**	7.6619	0.0040106	2.3968	0.012299
**2-Hydroxycaproic acid**	6.2949	0.0082348	2.0843	0.024439
**2,4-Diaminobutyric acid**	6.0161	0.0096412	2.0159	0.027718
**PC(18:1(9Z)/18:1(9Z))**	5.8788	0.010435	1.9815	0.029093

ANOVA for U373 cell line treated with paclitaxel 4.2 nM and/or etoposide 10 µg/mL				
	f.value	p.value	-log10(p)	FDR
**N-Acetylserotonin**	65.051	1.0802E-07	6.9665	1.0154E-05
**Sorbitol**	30.216	7.1011E-06	5.1487	0.00024643
**Adenosine monophosphate**	28.459	9.7124E-06	5.0127	0.00024643
**L-Arginine**	28.043	1.0487E-05	4.9794	0.00024643
**Succinylacetone**	24.205	2.2359E-05	4.6505	0.00042035
**N-Acetyl-L-alanine**	21.663	3.9135E-05	4.4074	0.00061312
**Glycerophosphocholine**	16.493	0.00014799	3.8298	0.0017648
**L-Phenylalanine**	16.441	0.00015019	3.8233	0.0017648
**Norepinephrine**	14.195	0.00029819	3.5255	0.0031145
**Guanosine monophosphate**	12.127	0.00060573	3.2177	0.0056939
**L-Tryptophan**	9.7647	0.00153	2.8153	0.013075
**L-Acetylcarnitine**	9.3266	0.0018475	2.7334	0.014472
**Phenylacetaldehyde**	8.834	0.0023004	2.6382	0.016634
**Guanosine 5'-diphosphate**	8.6121	0.0025459	2.5942	0.017094
**11a-Hydroxyprogesterone**	7.8704	0.0036194	2.4414	0.020993
**4-Hydroxyproline**	7.7936	0.0037581	2.425	0.020993
**L-Glutamic acid**	7.7728	0.0037966	2.4206	0.020993
**Inosine**	7.3763	0.0046294	2.3345	0.024176
**Thymidine**	6.9786	0.0056859	2.2452	0.02813
**Diaminopimelic acid**	6.3546	0.0079655	2.0988	0.037438
**L-Norleucine**	6.193	0.0087197	2.0595	0.039031
**Cyclic AMP**;	6.0719	0.0093389	2.0297	0.039903

Table S2. Collision-induced dissociation (CID) information

Type	Mass	Width	Collision	Charge State
**Base**	100.0000	2.00	20.00	1
**Base**	500.0000	4.00	20.00	1
**Base**	1000.0000	6.00	20.00	1
